# Noninvasive preoperative differential diagnosis of gallbladder carcinoma and xanthogranulomatous cholecystitis: A retrospective cohort study of 240 patients

**DOI:** 10.1002/cam4.4442

**Published:** 2021-11-27

**Authors:** Jianchun Xiao, Ruilin Zhou, Boyao Zhang, Binglu Li

**Affiliations:** ^1^ Department of General Surgery Peking Union Medical College Hospital Chinese Academy of Medical Science & Peking Union Medical College Beijing China; ^2^ Peking Union Medical College Chinese Academy of Medical Science & Peking Union Medical College Beijing China

**Keywords:** gallbladder carcinoma, imaging, noninvasive differential diagnosis, xanthogranulomatous cholecystitis

## Abstract

**Background:**

Xanthogranulomatous cholecystitis (XGC) is an extremely rare entity. Due to XGC’s clinical and radiological resemblance to gallbladder carcinoma (GBC), intraoperative frozen section during cholecystectomy is often performed to exclude the diagnosis of GBC. Our study is aiming to find a noninvasive indicator of XGC. To our knowledge, this is the largest XGC cohort ever studied.

**Methods:**

This study retrospectively collected clinical characteristics, serological tests, and imaging features of 150 GBC patients and 90 XGC patients. The diagnosis of these 150 GBC patients and 90 XGC patients was based on intraoperative frozen section histopathology. *T*‐test was utilized to compare differences between XGC and GBC. Receiver operating characteristic (ROC) curve was conducted and the area under the curve (AUC) was managed to evaluate the validity.

**Results:**

The carcinoembryonic antigen (CEA) level in blood tests was significantly elevated in GBC patients than in XGC patients (*p *= 0.007). The presence of submucosal hypo‐attenuated nodules (80% in XGC, 16% in GBC, *p *< 0.001), low density border (60% in XGC, 21% in GBC, *p *= 0.001), and nodular thickening in the bottom of the gallbladder with calcification (70% in XGC, 37% in GBC, *p *= 0.004) is significantly associated with XGC patients, whereas massive hilar infiltration (0% in XGC, 21% in GBC, *p *< 0.001), multiple lymph nodes in the hilar area (10% in XGC, 72% in GBC, *p *= 0.001), and gallbladder mucosal line continuity (50% in XGC, 95% in GBC, *p *= 0.002) are highly associated with GBC patients. The ROC curve was performed and the gallbladder mucosal line continuity (AUC = 0.708) and the AUC of low density border around the occupation (AUC = 0.654) showed a good prediction of XGC.

**Conclusions:**

Gallbladder mucosal line continuity and low density border around the occupation presented good indication value for the diagnosis of XGC. Our study proposed a noninvasive differential diagnosis method for XGC and GBC.

## Graphical abstract

Xanthogranulomatous cholecystitis (XGC) is a rare disease with limited clinical reports. Our study is currently the largest number of XGC patients included. We provided a potential non‐invasive diagnosis for XGC.

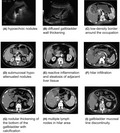

## 1 INTRODUCTION

Xanthogranulomatous cholecystitis (XGC), characterized by abnormal thickening of the gallbladder wall and inflammatory infiltration of nodular yellow mass, is a very rare but benign gallbladder disease. Histologically, the yellow mass is a mixture composed of foamy histiocytes, multinucleated giant cells, lymphocytes, and fibroblasts. XGC patients usually share similar symptoms with cholecystitis patients, and their imaging characteristics also present a certain degree of similarity with gallbladder carcinoma (GBC) patients’.^1^, ^2^, ^3^ It is often difficult to differentiate GBC from XGC, and the severe proliferative fibrosis surrounding the gallbladder might cause more confusion. The definitive diagnosis depends on pathologic examination after the performance of cholecystectomy and fine‐needle aspiration.^4^, ^5^ However, the clinical diagnosis before operation is often crucial for subsequent treatment and prognosis, which makes it important to differentiate these two diseases based on the clinical manifestations and imageology features. Moreover, this uncertainty in diagnosis may lead to unnecessary surgery, and thus inducing complications caused by surgery. Thus, it is crucial to develop a noninvasive method to differentiate XGC from GBC before the operation was performed.

In recent years, the utilization of radiology in differential diagnosis of XGC and GBC has gradually become a spotlight. Many studies were conducted to investigate the imaging features which might differentiate XGC from GBC. Diffuse gallbladder wall thickening, intramural nodules, intact gallbladder mucosa, and calculi are some feature computed tomography (CT) performances frequently mentioned.^6^, ^7^ However, the sample size of these studies is very limited. Therefore, we conducted this study to compare the imaging differences between XGC and GBC patients. We enrolled 90 XGC patients, which is largest cohort of XGC to our knowledge.

In this study, we conducted a retrospective study of the patients attending our hospital for either GBC or XGC in the past 12 years. We compared the clinical differences, serum biochemical tests, and imaging features of these patients. The aim of our study was to investigate the potential differentia in the radiological features between XGC and GBC, with the aim to elucidate a noninvasive differential diagnosis method in these two diseases, which could properly guide the follow‐up treatment.

## 2 MATERIALS AND METHODS

### 2.1 Study participant

From February 2008 to November 2018, 150 patients were diagnosed with GBC and 90 patients were diagnosed with XGC based on histopathological findings in our hospital. The inclusion criteria include: (1) the patient is diagnosed with either XGC or GBC and (2) the patient is previously hospitalized in our hospital. The exclusion criteria include: (1) the patients must not complicate with other cancer and (2) the patient has an unclear diagnose (i.e., with no pathology sent for examination). The criteria for XGC pathological diagnosis are: (1) foamy macrophages or macrophages with ceroid, bile, or iron; (2) also cholesterol clefts and multinucleated giant cells; (3) may be focal, nodular, or diffuse; and (4) may contain lymphocytes, plasma cells, foreign body giant cells, and neutrophils.

### 2.2 Evaluation of clinical indexes

The clinical characteristics and preoperative serum tests of these patients were collected and analyzed. The level of patients’ leukocyte, the absolute value of lymphocyte, and neutrophil count were detected by Sysmex XN and Siemens 2120. The ratio of albumin/globulin (ALB/GLB), the level of high‐density lipoprotein cholesterol (HDL‐C), free fatty acid (FFA), and high‐sensitivity C‐reactive protein (hsCRP) were detected by Beckman Coulter AU5800. The value of alpha‐fetoprotein (AFP), carcinoembryonic antigen (CEA), CA 19–9, CA 125, and CA242 was detected by Roche Cobas E801. And the abdominal ultrasound imaging and non‐contrast or contrast‐enhanced computed tomography (CT) scan imaging were also collected. The specific results are shown in Table S1. The study protocol was approved by the Ethical Committee of PUMCH.

### 2.3 Data analysis

All statistical analyses were performed employing SPSS version 13.0 (SPSS Inc.). Differences were evaluated using the independent samples *t*‐test, the χ2 test, the Mann–Whitney *U* test, or the Fisher's exact test, with statistically significant established at *p* values <0.05. The figures were drawn using the R ggplot2 package.

## 3 RESULTS

### 3.1 Clinical characteristics

Preliminary judgment of the patient's condition could be performed based on clinical manifestations. The clinical characteristics of all 240 enrolled patients are exhibited in Table 1. The most common clinical syndromes in these 240 patients include gallbladder stones, followed by a history of acute onset cholecystitis. Gallbladder stones were found more common in XGC patients (67.8%) than in GBC patients (37.3%). In addition, gallbladder polyps were found more common in GBC patients. There were four (4%) cases of biliary fistula/gallbladder perforation in XGC patients and none in GBC patients. Perforation and abscess formation were reported more in XGC patients (2 out of 90) than in GBC patients (0 out of 150). The percentage of biliary fistula/gallbladder perforation was significantly higher (*p* = 0.045) in XGC patients than in GBC patients, which is consistent with other reports.^3^, ^8^, ^9^, ^10^ Also, GBC patients presented a significantly higher percentage of chronic infection exposure (*p* < 0.001). Few statistical differences were encountered in jaundice, perforation and abscess formation, hyperlipidemia, and diabetes between these XGC patients and GBC patients. However, even these clinical features considered statistically significant were not particular in XGC patients, few differences in manifestations between patients with XGC and GBC could be revealed.

**TABLE 1:** Clinical characteristics of enrolled patients

Clinical characteristics	Xanthogranulomatous cholecystitis patients	Gallbladder carcinoma patients	*p* value
Number of patients	*N* = 90	*N* = 150	>0.05
Male number (percentage)	*N* = 54 (60%)	*N* = 59 (40%)	>0.05
Mean age (years old)	57.74	62.68	>0.05
History of acute onset cholecystitis	*N* = 57	*N* = 47	<0.001
Gallbladder stones	*N* = 61	*N* = 56	<0.001
Gallbladder polyps	*N* = 2	*N* = 14	0.028
Jaundice	*N* = 8	*N* = 18	>0.05
Biliary fistula/gallbladder perforation	*N* = 4	*N* = 0	0.045
Perforation and abscess formation	*N* = 2	*N* = 0	0.158
Chronic infection	*N* = 3	*N* = 40	<0.001
Hyperlipidemia	*N* = 10	*N* = 19	>0.05
Diabetes	*N* = 6	*N* = 33	>0.05

### 3.2 Serological tests

**FIGURE 1:**
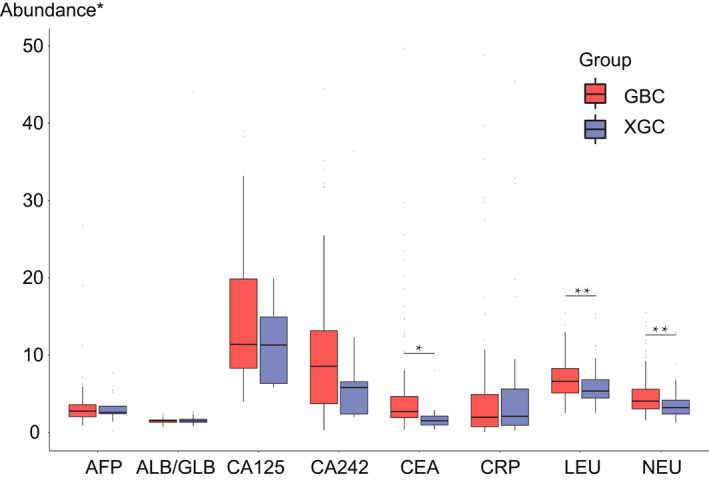
Boxplot of blood serum tests of xanthogranulomatous cholecystitis (XGC) group and gallbladder carcinoma (GBC) group. There was a significant difference in CEA level. Although all the tumor markers’ level in the GBC elevated, there was no significant difference between the two groups except for CEA. Extreme values were removed. There was also a significant difference in leukocyte and neutrophil absolute values. Both leukocyte and neutrophil absolute values were higher in GBC group than in XGC group. AFP, alpha‐fetoprotein; albumin/globulin (ALB/GLB), the ratio of blood albumin over blood globulin; CA125, carbohydrate antigen 125; CA242, carbohydrate antigen 242; CEA, carcinoembryonic antigen; CRP, C‐reactive protein; LEU, leukocyte; NEU, neutrophil. *Abundance unit: U/ml for AFP, CA125, CA242, and CEA; mg/L for CRP; ×10^9/L for LEU and NEU

Different blood tests were performed on all 240 patients. The specific results are shown in Table S1. Among 26 XGC patients and 110 GBC patients who took blood tumor marker tests, we found CEA (carcinoembryonic antigen) was significantly higher in GBC group (*p* = 0.007). Leukocyte and neutrophil absolute values also showed a significant difference between XGC and GBC groups, with *p* value equal to 0.004 and 0.001, respectively (Figure 1). However, there were few differences in other tumor markers such as AFP, CA 125, and CA242 between XGC group and GBC group. Besides, the ratio of ALB/GLB also showed few differences (Figure 1).

### 3.3 Ultrasonography

**FIGURE 2:**
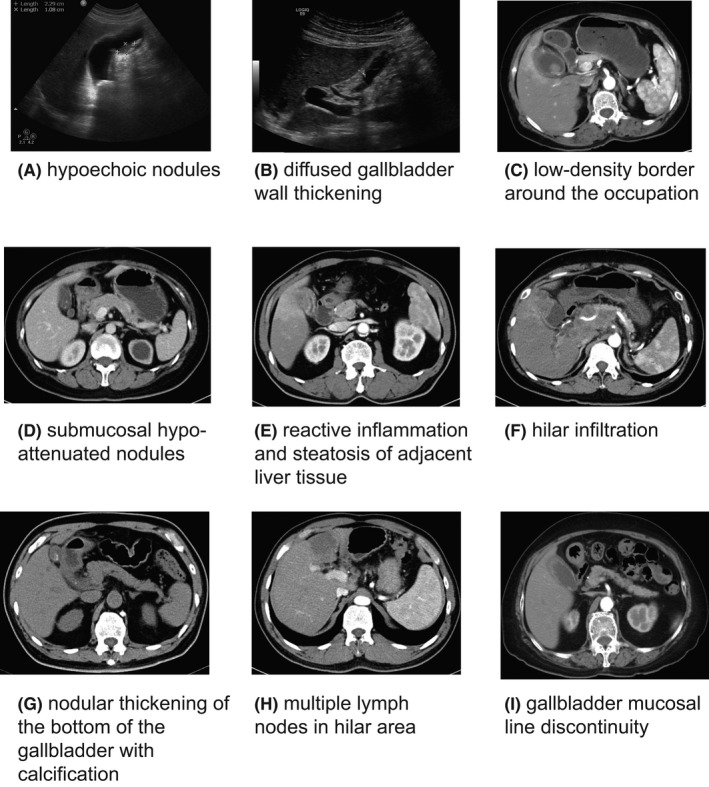
Xanthogranulomatous cholecystitis (XGC) and gallbladder carcinoma (GBC)’s characteristics in ultrasonography and computed tomography (CT) scan. Each of the disclosures in the figure has a corresponding figure. (A) hypoechoic nodules observed in XGC patients under ultrasound. (B) diffused gallbladder wall thickening observed in XGC patients under ultrasound. (C) low density border around the occupation. (D) submucosal hypo‐attenuated nodules. (E) reactive inflammation and steatosis of adjacent liver tissue. (F) hilar infiltration. (G) nodular thickening of the bottom of the gallbladder with calcification. (H) multiple lymph nodes in hilar area. (I) gallbladder mucosal line discontinuity

Ultrasonography was performed on 29 XGC patients and 57 GBC patients (Table 2). The sonographic discoveries included the presence of gallstones or sludge and moderate to marked focal or diffuse thickening of the gallbladder wall. The presence of hypoechoic nodules and the diffused gallbladder wall thickening could be observed occasionally, which is considered as a typical disclosure of XGC. Twenty‐four (82%) cases of hypoechoic nodules and 10 (17%) cases of hypoechoic nodules (Figure 2A) were observed on sonography in XGC and GBC, respectively, and the difference of which was significant (*p* < 0.001). The diffused gallbladder wall thickening (Figure 2B) on sonography was observed in 14 (48%) and 8 (14%) cases in XGC and GBC, respectively, and the difference of which was also significant (*p* = 0.002). The xanthogranulomatous nodules were taken as well‐defined hypoechoic areas on sonography.

**TABLE 2:** Ultrasonography results

Imaging characteristics	Xanthogranulomatous cholecystitis patients	Gallbladder carcinoma patients	*p* value
Number of patients	*N* = 29	*N* = 57	
Hypoechoic nodules (Figure 2A)	*N* = 24 (82%)	*N* = 10 (17%)	<0.001
Diffuse gallbladder wall thickening (Figure 2B)	*N* = 14 (48%)	*N* = 8 (14%)	0.02

### 3.4 Computed tomography

**FIGURE 3:**
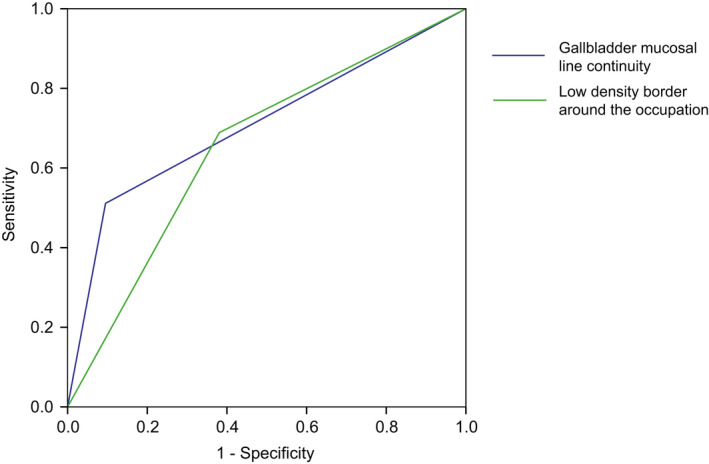
The Receiver operating characteristic (ROC) curve predicting xanthogranulomatous cholecystitis (XGC). The area under the curve (AUC) of gallbladder mucosal line continuity was 0.708 and the AUC of low density border around the occupation was 0.654

CT was performed on 20 XGC patients and 43 GBC patients. CT disclosures include––low density border around the occupation (Figure 2C), submucosal hypo‐attenuated nodules (Figure 2D), reactive inflammation and steatosis of adjacent liver tissue (Figure 2E), enlarged lymph nodes around the portal vein with nonspecific inflammatory reaction, hilar infiltration (Figure 2F), bile duct dilation, nodular thickening of the bottom of the gallbladder with calcification (Figure 2G), multiple high‐density shadows in the gallbladder (Figure 2H), multiple lymph nodes in hilar area, and gallbladder mucosal line continuity (Figure 2I). We believe it is the aggressiveness and metastasis of GBC led to a higher rate of reactive inflammation and steatosis of adjacent liver tissue (40% vs. 20%), hilar infiltration (21% vs. 0%), multiple lymph nodes in hilar area (72% vs. 10%), and gallbladder mucosal line discontinuity (95% vs. 50%). Cholelithiasis and choledocholithiasis seem to be associated more closely with XGC. Several imaging characteristics showed significant differences between XGC group and GBC group (Table 3). The gallbladder mucosal line continuity and low density border around the occupation showed great predictive value of XGC, with an AUC of 0.708 and 0.654, respectively (Figure 3). Moreover, our result presented a significant association between submucosal hypo‐attenuated nodules with XGC.

**TABLE 3:** Computed tomography results

Imaging characteristics	Xanthogranulomatous cholecystitis patients	Gallbladder carcinoma patients	*p* value
Number of patients	*N* = 20	*N* = 43	—
Low density border around the occupation (Figure 2C)	*N* = 12 (60%)	*N* = 9 (21%)	0.001
Submucosal hypo‐attenuated nodules (Figure 2D)	*N* = 16 (80%)	*N* = 7 (16%)	<0.001
Reactive inflammation and steatosis of adjacent liver tissue (Figure 2E)	*N* = 4 (20%)	*N* = 17 (40%)	0.053
Enlarged lymph nodes around the portal vein with nonspecific inflammatory reaction	*N* = 3 (15%)	*N* = 13 (30%)	0.582
Hilar infiltration (Figure 2F)	*N* = 0 (0%)	*N* = 9 (21%)	<0.001
Bile duct dilation	*N* = 7 (35%)	*N* = 21 (49%)	0.543
Nodular thickening of the bottom of the gallbladder with calcification (Figure 2G)	*N* = 14 (70%)	*N* = 16 (37%)	0.004
Multiple high‐density shadows in the gallbladder (Figure 2H)	*N* = 9 (45%)	*N* = 18 (42%)	0.450
Multiple lymph nodes in hilar area	*N* = 2 (10%)	*N* = 31 (72%)	0.001
Gallbladder mucosal line discontinuity (Figure 2I)	*N* = 10 (50%)	*N* = 41 (95%)	0.002

## 4 DISCUSSION

XGC, characterized by infiltration of inflammatory cells, foamy cells, and fibroblasts, is a clinically rare disease. It mainly occurs in older people, usually people at the age of 60–70, and with men and women equally suffering. Patients with XGC also present different manifestations. XGC is often misdiagnosed as GBC due to their clinical similarity. Usually, gallbladder cancer cannot be completely ruled out until the biopsy specimens of the surgical sample are sent for pathological examination. Thus, we conducted this study to develop a noninvasive method to differentiate XGC from GBC.

In our study, we found differentiations in clinical symptoms and biochemical tests between XGC and GBC patients. Our results showed that the perforation and abscess formation was more common in XGC patients than in GBC patients, which is consistent with other case reports.^8^, ^11^, ^12^ In addition to clinical manifestations, serological tests also showed significant differences between XGB patients and GBC patients. As for the serum levels of the most commonly used gastrointestinal tumor markers such as CA19‐9, CA 125, AFP, CA 242, and CEA, our study presented a quite interesting result. CA19‐9, which was documented as an upper gastrointestinal malignancies closely associated tumor marker,^13^ showed no significant difference between XGC and GBC. What is more, CA125, CA19‐9, CA125, and CA242, which are all reported as diagnostic markers in carcinoma of the gallbladder,^14^, ^15^ did not show a significant difference between XGC and GBC patients, either. However, CEA was the only tumor marker that showed a significant difference between GBC and XGC patients (higher in XGC patients). Although it was reported that CA19‐9 concentration is superior to CEA in judging the necessity of surgical removal of advanced gallbladder cancer,^16^ as well as in prediction of disease progression of GBC patients,^17^, ^18^ our result showed that CEA showed an advantage in differentiating GBC and XGC. Thus, CEA presented potential in differentiating XGC and GBC.

Besides the difference in biochemical tests, we discovered several radiological characteristics to rule out the possibility of GBC, especially in CT scans. Our result showed that hypoechoic nodules and diffuse gallbladder wall thickening were more associated with XGC. Our finding is consistent with that of Zhang's ^19^ and Li's studies.^19^, ^20^ Zhang's study showed that hypoechoic nodules were found in 90.3% (28 out of 31) of XGC patients and 11.5% (6 out of 52) of GBC patients. Moreover, Li's study also found the presence of hypoechoic nodules in 58.8% of XGC patients (10 out of 17) and 25.6% of GBC patients (11 out of 43, *p* = 0.015). Both of these two studies showed a significantly higher presence of hypoechoic nodules in XGC than in GBC, which is consistent with our results. But our study has a relatively larger cohort (90 XGC patients) than Zhang's (contained only 31 XGC patients) and Li's studies (contained only 17 XGC patients). Making the results more reliable. What is more, according to Lee's research, diffuse gallbladder wall thickening was more likely to be observed in XGC patients (72%) than in GBC patients (14 patients, 25.0%) (*p* < 0.001).^4^ Consistently, our study showed that there was a significant difference in gallbladder wall thickening between XGC and GBC patients (*p* = 0.02). Once again, our study has a larger cohort than Lee's study. Thus the thickening of gallbladder could also be considered as an imaging difference between XGC and GBC. In addition, our findings of gallbladder mucosal line continuity and low density border around the occupation have not been reported in other researches. This feature may be considered as a novel noninvasive differential method for XGC and GBC.

Our study provided a noninvasive method in distinguishing XGC from GBC. By taking CT scan, we could distinguish most of the XGC cases from GBC and thus avoid the practice of biopsy. Furthermore, the risks in undergoing biopsy are avoided using this noninvasive evaluation and medical expenses are saved. This noninvasive evaluation is not only safe but also money saving for patients. In addition, to our knowledge, this is the largest XGC cohort ever studied. As far as we know, other researches studies enrolled a relatively small number of patients, from 20 to at most 30.^21^ Our study enrolled a total of 90 XGC patients, making our results more reliable.

Our study has some limitations. Since few patients in our study underwent MRI or PET‐CT, we did not perform statistical analysis on MRI and PET‐CT’s diagnostic value in XGC. The inclusion of MRI and PET‐CT may enrich the results.

## 5 CONCLUSIONS

Our study provided a noninvasive method for differential diagnosis of XGC and GBC. Gallbladder mucosal line continuity and low density border around the occupation presented good indication value for the diagnosis of XGC. Our study may shed a new light on the diagnosis of XGC and its possible pathophysiology.

## CONFLICT OF INTEREST

The authors declare no conflict of interest.

## AUTHOR CONTRIBUTIONS

Jianchun Xiao designed the experiments and collected the data. Ruilin Zhou conducted the literature research, statistical analysis, and writing most of the manuscript. Boyao Zhang contributed to the literature research and helped to collect samples and clinical data. Binglu Li revised the manuscript. All authors read and approved the final manuscript.

## ETHICS APPROVAL AND CONSENT TO PARTICIPATE

This study was approved by the Ethics Review Board at the Institute of Basic Medical Sciences, Chinese Academy of Medical Sciences, and informed consent was acquired from each patient. All experiments were performed in accordance with relevant guidelines and regulations.

## Supporting information

Table S1

## Data Availability

Not applicable.
